# Implicações Cardiovasculares em Pacientes Infectados com Covid-19 e a Importância do Isolamento Social para Reduzir a Disseminação da Doença

**DOI:** 10.36660/abc.20200243

**Published:** 2020-05-22

**Authors:** Juliana Alves Costa, Juliana de Almeida Silveira, Sara Cristine Marques dos Santos, Patrícia Pereira Nogueira

**Affiliations:** 1 Universidade de Vassouras Rio de JaneiroRJ Brasil Universidade de Vassouras – Cardiologia,Rio de Janeiro, RJ – Brasil

**Keywords:** Coronavirus, COVID-19, Infecções por Coronavirus/prevenção e controle, Isolamento Social, Disseminação da Doença

## Abstract

Os sintomas respiratórios, principalmente o desenvolvimento de quadros de síndrome do desconforto respiratório agudo grave, dominam a discussão e as preocupações iniciais da população e dos profissionais de saúde. Entretanto, o sistema cardiovascular é bastante afetado por essas condições e, muitas vezes, é o responsável por complicações e mortalidade desses pacientes. Com o objetivo de mostrar as implicações cardiovasculares em pacientes infectados pela COVID-19 e a importância do isolamento social como alternativa de frear a disseminação da doença, foi realizada uma revisão da literatura com base em 37 artigos, nos idiomas inglês, português e espanhol, disponíveis na plataforma Scielo e PubMed. Os resultados mostraram que complicações cardíacas associadas à infecção pela COVID-19 são semelhantes às produzidas por: síndrome respiratória aguda grave (SARS), síndrome respiratória do Médio Oriente (MERS) e influenza. Contudo, a COVID-19 apresenta uma contaminação muito maior e mais rápida e, ao contrário da gripe por influenza, ainda não existe vacina disponível ou tratamento. Diante disso, o isolamento social passa a ser uma ferramenta que pode reduzir e achatar a curva de casos incidentes e assim preservar as pessoas que se enquadrem no grupo de risco, diminuindo as chances de quadros graves da doença, possíveis óbitos e o colapso no sistema de saúde do país.

## Introdução

O coronavírus se trata de um vírus pertencente à família *Coronaviridae*, causadores de uma gripe simples até doenças que podem causar risco maior à saúde da população. O novo coronavírus, causador da pandemia em 2020, recebeu a denominação SARS-CoV-2 pela Organização Mundial da Saúde (OMS), e a doença que ele provoca tem a denominação COVID-19.^1^ Primeiramente foi detectado em dezembro de 2019 em Wuhan, na China. No entanto, em virtude de seu alto poder de disseminação, diversos países confirmaram a presença de casos alóctones em meados de janeiro de 2020. No Brasil, confirmou-se o primeiro caso no dia 26 de fevereiro de 2020.^2,3^

Até o aparecimento do SARS-CoV-2, estavam descritas duas outras epidemias causadas por coronavírus: o SARS-CoV-1, causador da síndrome respiratória aguda grave (SARS), em 2002; e MERS-CoV, causador da síndrome respiratória do Médio Oriente (MERS), em 2012.^4^ A fisiopatologia do SARS-CoV-2 demostrou-se semelhante à do SARS-CoV-1, pois apresentam lesões pulmonares agudas devido à inflamação agressiva iniciada pela replicação viral. A infecção por SARS-CoV-2 pode causar aumento da secreção de interleucinas pró-inflamatórias e de interferon-gama (IFN-γ) que provocam as lesões pulmonares.^5^

O Brasil, assim como outros países, passa pelo processo de transição demográfica que tem como principal efeito o envelhecimento da população. Assim, as doenças do sistema circulatório aparecem como principal causa de mortalidade da população. Associando essa informação aos estudos recentes das implicações cardiovasculares e seu agravamento pelo SARS-CoV-2, fica evidente que medidas de prevenção e controle que diminuam riscos de contaminação e infecção são importantes ferramentas na diminuição de casos graves da doença e eventuais óbitos.

O presente artigo tem como objetivo relacionar a atual pandemia de COVID-19 com as implicações cardiovasculares, mostrando a importância do isolamento social como medida de prevenção e controle de disseminação da doença e preservação do Sistema de Saúde do país.

## Material e métodos

Revisão da literatura com base em 37 artigos, nos idiomas inglês, português e espanhol, disponíveis na plataforma Scielo e PubMed, referentes as implicações cardiovasculares nos pacientes infectados pelo covid-19, importância do isolamento social como medida de prevenção e controle de disseminação da doença e preservação do sistema de saúde do país.

### Panorama geral da COVID-19

A pandemia da COVID-19, assim como as epidemias prévias de outros coronavírus (SARS e MERS) e a pandemia de 2009 (H1N1), traz consequências graves nos modelos de saúde, econômicos e sociais de toda a população mundial.

A transição demográfica, apesar de ocorrer de forma diferenciada entre os países, de modo geral, caracteriza-se pelo aumento da população idosa em comparação às demais faixas etárias, pois cresce cerca de 4% ao ano. Fatores como diminuição da fecundidade, redução da mortalidade infantil e da mortalidade geral, melhorias na atenção à saúde da população, desenvolvimento tecnológico no tocante ao diagnóstico e tratamento das doenças também corroboram para o quadro demográfico atual.^6^

Concomitante ao aumento do número de idosos, ocorre uma transição epidemiológica, com crescimento da proporção das doenças no aparelho circulatório, diabetes melito, neoplasias, agravos por causas externas e doenças do aparelho respiratório.^7^ Estudos indicam que uma maior frequência de comorbidades está comumente relacionada à idade mais avançada.

A taxa de mortalidade da COVID-19 pode ser nove vezes maior entre pessoas com alguma doença crônica, quando comparada à de pacientes sem patologia preexistente. Dados disponibilizados pela Organização Mundial da Saúde (OMS) em fevereiro demonstram que no grupo de infectados sem comorbidade apenas 1,4% morreu. Já entre os pacientes com alguma doença cardiovascular, por exemplo, o índice chegou a 13,2%. Considerando todos os pacientes infectados, a letalidade foi de 3,8%, mas vale ressaltar que, em função do andamento da pandemia, novos dados estatísticos vêm sendo adicionados aos estudos.

A forma severa da doença foi observada nos pacientes mais velhos^8,9^ que apresentavam um número mais significativo de condições comórbidas comparados aos pacientes não graves. Esses resultados sugerem que idade e comorbidades associadas podem ser um dos fatores de risco para pacientes críticos. Além disso, idosos e pacientes imunossuprimidos podem manifestar sintomas atípicos e outras maneiras de apresentação, incluindo pneumonia leve, moderada e grave, e em casos mais graves, síndrome respiratória aguda grave, sepse, choque séptico e morte.^4^

Em um relato de caso de 138 pacientes hospitalizados com COVID-19, 16,7% dos pacientes desenvolveram arritmia e 7,2% sofreram lesão cardíaca aguda, além de outras complicações relacionadas com a COVID-19. Relatórios publicados indicam casos de insuficiência cardíaca de início agudo, infarto do miocárdio, miocardite e parada cardíaca.^10^Além disso, foram encontrados casos de dano miocárdico, com aumento de troponina I, dano cardíaco agudo, choque e arritmia.^11,12^

Na fase aguda dos quadros virais graves, não somente da COVID-19, mas também em outras coronoviroses, o paciente pode apresentar taquicardia, hipotensão, bradicardia, arritmias e morte súbita. Alterações eletrocardiográficas e aumento de troponina sinalizam acometimento miocárdico na forma de miocardite.^11,12^

Estudos de coortes publicadas até o momento mostram taxas de insuficiência cardíaca aguda, choque e arritmia de 7,2%, 8,7% e 16,7%, respectivamente. O acometimento cardiovascular decorre devido a um descompasso entre o aumento da demanda metabólica/inflamatória desencadeado pelo vírus e uma reserva cardíaca reduzida. O estado inflamatório torna o ambiente mais propenso a fenômenos trombóticos. Sendo assim, a recomendação tem sido de que as medicações de uso crônico dos pacientes sejam mantidas, sendo a sua retirada/substituição avaliada em nível individual e de acordo com as diretrizes vigentes até o momento. Vale ressaltar que novas recomendações podem surgir à medida que saem novos trabalhos em andamento.^13,14^

As doenças crônicas, tais como hipertensão, diabetes, doenças do sistema respiratório, doenças cardiovasculares e suas condições de suscetibilidade, compartilham com as doenças infecciosas alguns estados padronizados, como o estado pró-inflamatório e a atenuação da resposta imune inata. O diabetes, por exemplo, ocorre, em parte, porque o acúmulo de células imunes inatas ativadas nos tecidos metabólicos leva à liberação de mediadores inflamatórios, especialmente IL-1β e TNFα, que promovem resistência à insulina e danos às células β.^15^ Além disso, os distúrbios metabólicos podem levar à depressão da função imunológica, prejudicando a função dos macrófagos e linfócitos,^16^ o que pode tornar os indivíduos mais suscetíveis a complicações e agravos da COVID-19.^9^

Muitos dos pacientes mais velhos que ficam gravemente doentes têm evidências de doenças subjacentes, tais como doença cardiovascular, doença hepática, doença renal ou tumores malignos.^17-19^ Esses pacientes geralmente morrem de suas comorbidades originais, portanto, a avaliação precisa de todas as comorbidades originais dos indivíduos com COVID-19 deve ser rigorosamente analisada e considerada no plano terapêutico individualizado.

Outros trabalhos acrescentam que a insuficiência respiratória agravada pelo SARS-CoV-2 ocorre em virtude de danos alveolares maciços. Esse vírus é capaz de infectar células epiteliais respiratórias humanas por meio de uma interação entre a proteína S viral e o receptor da enzima 2 de conversão da angiotensina nas células humanas. Embora, na literatura, haja evidências de que a presença de infecções pulmonares graves possa afetar o prognóstico a longo prazo dos indivíduos cardiopatas, não existem dados que confirmem que pacientes recuperados da infecção por COVID-19 irão experimentar efeitos a longo prazo.^20,21^

Assim, não apenas capaz de causar pneumonia, a COVID-19 também pode provocar danos a outros órgãos, e os pacientes acabam morrendo por insuficiência de múltiplos órgãos, choque, síndrome do desconforto respiratório agudo, insuficiência cardíaca, arritmias e insuficiência renal.^22^ As possíveis lesões de múltiplos órgãos e sua proteção e prevenção devem ser monitoradas no tratamento da COVID-19.^23^Nesses pacientes críticos, as medidas de proteção necessárias incluem ventilação mecânica, glicocorticoides, antivirais, tratamentos sintomáticos e terapia antichoque.

Outro fator importante seria a maneira de estimar a capacidade de transmissibilidade de um vírus por meio do cálculo de seu número reprodutivo (R0), o que representa uma medida de sua taxa de ataque, ou seja, traduz o número de infecções secundárias que ocorrem a partir de um indivíduo infectado em uma população suscetível. Estudos preliminares apontavam que este novo coronavírus, responsável pela COVID-19, estaria associado a taxas de R0 de 1,5 a 3,5, sendo os dados mais recentes sugerindo um R0 de 4,08 (i. e., para cada caso, em média, haveria quatro novos indivíduos infectados).^7^

Por apresentar alto potencial de disseminação,^1^ e sabendo que se trata de um vírus RNA, envelopado e com contaminação por gotículas respiratórias ou contato, as medidas de higiene devem ser reforçadas e colocadas em prática. Estas são: lavar as mãos com água e sabão para destruir a estrutura morfológica do vírus, usar álcool 70%, cobrir a boca ao tossir ou espirrar para evitar que as partículas virais se disseminem pelo ambiente, evitar aglomerações e manter-se em local bem ventilado.^3,4^

De acordo com a literatura, o período médio de incubação por coronavírus é de 5 dias, com intervalos que chegam a 12 dias. Dados preliminares do SARS-CoV-2 sugerem que a transmissão possa ocorrer mesmo sem o aparecimento de sinais e sintomas.^4,5^

Quando não há complicações, os sintomas apresentados consistem em febre, tosse seca e sensação de cansaço, podendo haver ocorrência de coriza e congestão nasal, dor de garganta e diarreia. Além disso, grande parte dos infectados é assintomática (cerca de 80%) e se recupera sem a necessidade de tratamento especial, enquanto 1/6 dos pacientes pode evoluir de forma grave, cursando com dificuldade para respirar.^24,25^

Diante da situação atual, é de extrema importância que a população se conscientize e faça isolamento domiciliar, apresentando-se sintomática ou não, com o objetivo de diminuir o número de pessoas contaminadas e atrasar a transmissão comunitária disseminada, para que o sistema de saúde público consiga atender a todos.^26^ Caso contrário, o crescimento exponencial da doença poderá colapsar este sistema, levando a óbito aqueles mais fragilizados. Já as pessoas que tiverem a necessidade de circular em locais públicos, por motivo de trabalho ou força maior, devem adotar medidas preventivas.^27^

**Figura 1:**
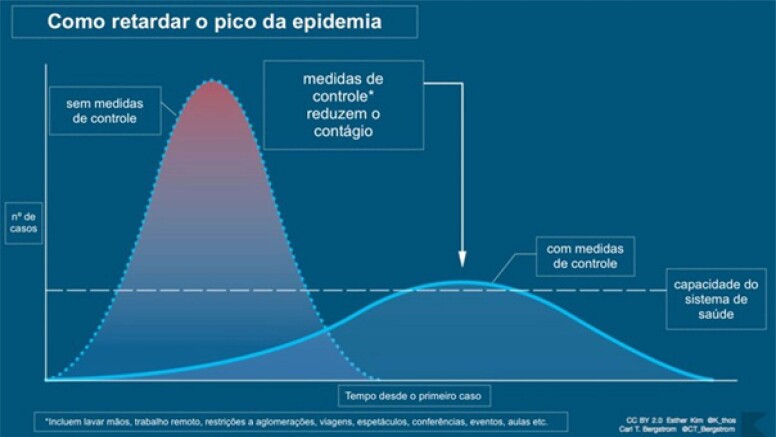
– *Curva de disseminação do vírus.*

Desacelerar a disseminação do vírus para que o número de casos se espalhe ao longo do tempo em vez de haver picos no início é uma das formas de achatar a curva epidêmica e evitar que o sistema púbico de saúde entre em colapso e, como consequência, muitas pessoas acabem indo a óbito (Figura 1). Uma transmissão controlada da doença reduz a pressão sobre o sistema de saúde e aumenta a capacidade de cuidados não apenas para os pacientes contaminados por coronavírus, mas também para aqueles que necessitam de atendimento médico por outras enfermidades.

O isolamento domiciliar é uma maneira de permanecer em casa tomando alguns cuidados que diminuem o risco de transmitir infecções respiratórias, como a provocada pelo coronavírus (COVID-19). Esses cuidados especiais impedem que o contato das secreções respiratórias de uma pessoa que possa estar infectada pelo coronavírus entrem em contato com outras.^19^ Devem ficar nessa circunstância pessoas que positivaram no teste para o referido vírus ou que estão sob suspeita. O ideal é que o indivíduo fique sozinho em um quarto, ou em um cômodo da casa adaptado como quarto, se possível com um banheiro privativo^28,29^ As portas do quarto devem ficar fechadas o tempo todo, mas as janelas devem ficar abertas para que o ambiente fique bem ventilado. O paciente só deve sair desse cômodo isolado em caso de necessidade.^30-32^

Diante disso, o isolamento social e as medidas de prevenção são necessários para evitar, principalmente, que os idosos se infectem e venham a agravar doenças preexistentes, complicando o quadro de saúde e levando a sinais e sintomas que muitas vezes podem ser fatais.^33,34^ Quando uma doença se espalha de maneira rápida, os serviços ficam superlotados, não há leitos, máscaras, médicos, respiradores e outros equipamentos suficientes para quem precisa, e isso não só para a COVID-19, mas para qualquer outra enfermidade que requeira que o paciente procure o sistema de saúde. É preciso evitar que o sistema entre em colapso.^35^

## Considerações finais

Há um consenso entre os autores de que o grupo de maior risco para desenvolver a forma mais grave, podendo evoluir para óbito, inclui idosos e indivíduos que apresentem as comorbidades mais prevalentes, dentre elas as doenças cardiovasculares.^36^

O crucial não é a gravidade da doença em si, mas a capacidade de dar atenção a todos os infectados no momento em que eles precisarem. Quanto mais se achata a curva de transmissão ao longo do tempo, menor a sobrecarga no sistema de saúde e maior a probabilidade de que ele dê conta da demanda epidêmica – o que evidencia a importância do isolamento social como medida de prevenção e controle de disseminação da doença e preservação do sistema de saúde do país.
