# Supplementary material for: Implicações Cardiovasculares em Pacientes Infectados com Covid-19 e a Importância do Isolamento Social para Reduzir a Disseminação da Doença
Source: Arq Bras Cardiol. 2020 May 22;114(5):834–8. [Article in Portuguese] doi: 10.36660/abc.20200243 (PMC8386993; doi:10.36660/abc.20200243)
Supplement: Translation

# Translation: Cardiovascular Implications in Patients Infected with Covid-19 and the Importance of Social Isolation to Reduce Dissemination of the Disease

**Authors:** Juliana Alves Costa, Juliana de Almeida Silveira, Sara Cristine Marques dos Santos, Patrícia Pereira Nogueira

**Affiliations:** 1 Brazil Universidade de Vassouras – Cardiologia, Rio de Janeiro, RJ – Brazil

**Keywords:** Coronavirus, COVID-19, Infecções por Coronavirus/prevention and control, Social Isolation, Diseases Dissemination

## Abstract

Respiratory symptoms, especially the development of severe acute respiratory distress syndrome, dominate the discussion and initial concerns of the population and health professionals. However, the cardiovascular system is greatly affected by these conditions and is often responsible for complications and mortality of these patients. In order to show the cardiovascular implications in patients infected with COVID-19 and the importance of social isolation as an alternative to curb the spread of the disease, a literature review was carried out based on 37 articles, in English, Portuguese and Spanish, available on Scielo and PubMed. The findings showed that cardiac complications associated with COVID-19 infection are similar to those produced by: severe acute respiratory syndrome (SARS), Middle East respiratory syndrome (MERS) and influenza. However, COVID-19 has a much greater and faster contamination and, unlike influenza, there is no vaccine or treatment available yet. In view of this, social isolation becomes a tool that can reduce and flatten the curve of cases and thus protect the people at higher risk, decreasing the chances of serious conditions related to the disease, potential deaths and the collapse of the country’s health system.

## Introduction

Coronavirus is a virus belonging to the *Coronaviridae* family, causing simple flu to diseases that can cause greater risks to the population’s health. The novel coronavirus, which caused the 2020 pandemic, received the name SARS-CoV-2 by the World Health Organization (WHO), and the disease it causes has the name: COVID-19.^1^ It was first detected in December 2019 in Wuhan, China. However, due to its high dissemination power, several countries confirmed the presence of allochthonous cases in mid-January 2020. In Brazil, the first case was confirmed on February 26, 2020.^2,3^

Until the appearance of SARS-CoV-2, two other epidemics caused by coronavirus were described: SARS-CoV-1, which causes severe acute respiratory syndrome (SARS), in 2002; and MERS-CoV, which caused the Middle East respiratory syndrome (MERS), in 2012.^4^ The pathophysiology of SARS-CoV-2 was similar to that of SARS-CoV-1, as they present acute lung injuries due to the aggressive inflammation initiated by viral replication. SARS-CoV-2 infection can cause increased secretion of pro-inflammatory interleukins and interferon-gamma (IFN-γ) that cause lung damage.^5^

Brazil, like other countries, is going through the process of demographic transition that has the aging of the population as its main effect. Thus, diseases of the circulatory system appear as the main cause of mortality in the population. By associating this information with recent studies of cardiovascular implications and their worsening by SARS-CoV-2, it is evident that prevention and control measures that reduce risks of contamination and infection are important tools in the reduction of severe cases of the disease and potential deaths.

This article relates the current pandemic of COVID-19 with the cardiovascular implications, showing the importance of social isolation as a measure to prevent and control the spread of the disease and preserve the country’s Health System.

## Material and methods

Literature review based on 37 articles, in English, Portuguese and Spanish, available on Scielo and PubMed, referring to the cardiovascular implications in patients infected with Covid-19, the importance of social isolation as a measure of prevention and control of disease spread and preservation of the country’s health system.

### Overview of COVID-19

The COVID-19 pandemic, as well as previous epidemics of other coronaviruses (SARS and MERS) and the 2009 pandemic (H1N1), have serious consequences for the health, economic and social models of the entire world population.

Although demographic transition occurs differently from country to country, in general, is characterized by an increase in the elderly population compared to other age groups, as it grows about 4% per year. Factors such as reduced fertility, reduced infant mortality and general mortality, improvements in health care for the population, technological development with regard to the diagnosis and treatment of diseases also corroborate the current demographic scenario.^6^

Concomitantly with the increase in the number of elderly people, there is an epidemiological transition, with an increase in the proportion of circulatory diseases, diabetes mellitus, neoplasms, diseases due to external causes and diseases of the respiratory system.^7^ Studies show that a higher frequency of comorbidities is commonly related to older age.

The mortality rate of COVID-19 can be nine times higher among people with some chronic disease compared to that of patients without any pre-existing pathology. Data provided by the World Health Organization (WHO) in February show that in the group of infected people without comorbidities, only 1.4% died. Among patients with some cardiovascular disease, for example, the rate reached 13.2%. Considering all infected patients, lethality was 3.8%, but it is worth mentioning that, due to the progress of the pandemic, new statistical data has been added to the studies.

The severe form of the disease was observed in older patients^8,9^ who had a more significant number of comorbid conditions compared to non-severe patients. These findings suggest that age and associated comorbidities may be one of the risk factors for critically ill patients. Besides, the elderly and immunosuppressed patients may manifest atypical symptoms and other forms of presentation, including mild, moderate and severe pneumonia and, in more severe cases, severe acute respiratory syndrome, sepsis, septic shock and death.^4^

In a case report of 138 patients hospitalized with COVID-19, 16.7% of patients developed arrhythmia and 7.2% suffered acute cardiac injury, in addition to other complications related to COVID-19. Published reports indicate cases of acute onset heart failure, myocardial infarction, myocarditis and cardiac arrest.^10^ Moreover, cases of myocardial damage, with increased troponin I, acute cardiac damage, shock and arrhythmia, were found.^11,12^

In the acute phase of severe viral conditions, not only in COVID-19, but also in other Coronavirus illnesses, the patient may present tachycardia, hypotension, bradycardia, arrhythmias and sudden death. Abnormal findings on electrocardiograms and increased troponin signal myocardial involvement in the form of myocarditis.^11,12^

Cohort studies published to date show rates of acute heart failure, shock and arrhythmia of 7.2%, 8.7% and 16.7%, respectively. Cardiovascular involvement is due to a mismatch between the increased metabolic/inflammatory demand triggered by the virus and reduced cardiac reserve. The inflammatory state makes the environment more prone to thrombotic phenomena. Therefore, the recommendation has been that patients’ chronic medications should be maintained, with their withdrawal/replacement being assessed on an individual level and in accordance with the guidelines in force so far. It is worth noting that new recommendations may emerge as new studies in progress come out.^13,14^

Chronic diseases, such as hypertension, diabetes, diseases of the respiratory system, cardiovascular diseases and their conditions of susceptibility, share some standardized states with infectious diseases, such as pro-inflammatory state and the attenuation of innate immune response. Diabetes, for example, occurs partly because the accumulation of innate immune cells activated in metabolic tissues leads to the release of inflammatory mediators, especially IL-1β and TNFα, which promote insulin resistance and damage to β cells.^15^ Moreover, metabolic disorders can lead to depression of the immune function, impairing the function of macrophages and lymphocytes,^16^ which can make individuals more susceptible to complications and aggravations of COVID-19.^9^

Many of the older patients who become seriously ill have evidence of underlying diseases, such as cardiovascular diseases, liver diseases, kidney diseases or malignant tumors.^17-19^ These patients usually die from their original comorbidities. Therefore, the accurate assessment of all original comorbidities of individuals with COVID-19 must be rigorously analyzed and considered from an individualized therapeutic perspective.

Other studies add that the respiratory failure aggravated by SARS-CoV-2 occurs due to massive alveolar damage. This virus is capable of infecting human respiratory epithelial cells through an interaction between the viral S protein and the angiotensin-converting enzyme 2 receptor in human cells. Although there is evidence in the literature that the presence of severe lung infections can affect the long-term prognosis of individuals with heart diseases, there is no data to confirm that patients recovered from COVID-19 infection will experience long-term effects.^20,21^

Thus, not only capable of causing pneumonia, COVID-19 can also cause damage to other organs, and patients end up dying from multiple organ failure, shock, acute respiratory distress syndrome, heart failure, arrhythmias and kidney failure.^22^ Potential injuries to multiple organs and their protection and prevention should be monitored in the treatment of COVID-19.^23^ In these critical patients, the necessary protective measures include mechanical ventilation, glucocorticoids, antivirals, symptomatic treatments and shock therapy.

Another important factor would be the approach to estimating the transmissibility of a virus by calculating its reproductive number (R0), which represents a measure of its attack rate, that is, it translates the number of secondary infections that occur from an infected individual in a susceptible population. Preliminary studies pointed out that this new coronavirus, responsible for COVID-19, would be associated with R0 rates of 1.5 to 3.5, with the most recent data suggesting an R0 of 4.08 (i.e., for each case, on average , there would be four new infected individuals).^7^

As it has a high potential for dissemination1 and, knowing that it is an RNA virus, enveloped and contaminated by respiratory droplets or contact, hygiene measures must be improved and put into practice. These are: washing our hands with soap and water to destroy the morphological structure of the virus, using 70% alcohol-based hand sanitizer, covering our mouth when coughing or sneezing to prevent viral particles from spreading through the environment, avoiding crowds and staying in a well-ventilated area.^3,4^

According to the literature, the average incubation period for coronavirus is 5 days, with intervals that can be as long as 12 days. Preliminary data for SARS-CoV-2 suggest that transmission may occur even without the appearance of signs and symptoms.^4,5^

When there are no complications, the symptoms consist of fever, dry cough and tiredness. Runny nose and nasal congestion, sore throat and diarrhea may also occur. Furthermore, most of those infected are asymptomatic (about 80%) and recover without requiring any special treatment, while 1/6 of the patients can progress severely, with breathing issues.^24,25^

In view of the current situation, it is extremely important for the population to act conscientiously and stay at home, whether symptomatic or not, with the aim of reducing the number of infected people and delaying the disseminated community transmission, so that the public health system may be able to serve everyone.^26^ Otherwise, the exponential growth of the disease may break the health system, leading to the death of the most fragile ones. Those who need to hang around in public places, due to work or force majeure, should take preventive measures.^27^

Slowing down the spread of the virus so that the number of cases spreads over time instead of having peaks in the beginning is one of the ways to flatten the epidemic curve and prevent the public health system from collapsing and, as a consequence, many people end up dying (Figure 1). Controlled disease transmission reduces pressure on the health system and increases the capacity of taking care not only of patients infected with coronavirus, but also those requiring medical care due to other illnesses.

**Figura 1:** – *Virus spread curve.*

Staying at home is intended to provide a means of precaution to reduce the risk of transmitting respiratory infections, such as that caused by the coronavirus (COVID-19). These special precautions prevent the contact of respiratory secretions of a person who may be infected with the coronavirus from coming into contact with others.^19^ People who have tested positive for the said virus or are under suspicion should remain at home. Ideally, the individual should be alone in a bedroom or in a room that may temporarily serve as a bedroom, with a private bathroom if possible.^28,29^ The bedroom doors should be closed all the time, but the windows should be open so that the area may stay well ventilated. The patient should only leave this isolated room if necessary.^30-32^

Therefore, social isolation and preventive measures are necessary to prevent, mainly, that the elderly get infected and worsen preexisting diseases, complicating their health situation and leading to signs and symptoms that can often be fatal.^33,34^ When a disease spreads quickly, services are overcrowded, there are not enough beds, masks, doctors, ventilators and other equipment for those in need, and this is not only for COVID-19, but for any other illness that requires the patients to seek health care. The system must be prevented from collapsing.^35^

## Final considerations

There is consensus among the authors that the group at higher risk for developing the most severe form, which may lead to death, includes the elderly and individuals who have the most prevalent comorbidities, including cardiovascular diseases.^36^

The crucial thing is not the severity of the disease itself, but the ability to care for all those infected when they need it. The more the transmission curve is flattened over time, the less the burden on the health system and the greater the likelihood that it will meet the epidemic demand — which highlights the importance of social isolation as a measure for preventing and controlling the spread of the disease and preserving the country’s health system.
